# Extracellular vesicles in head and neck cancer: mediators of oncogenesis, immune evasion, and therapy resistance

**DOI:** 10.3389/fimmu.2025.1642639

**Published:** 2025-09-19

**Authors:** Jillian Dean, Tobias Niederegger, Cosima C. Hoch, Bhagvat Maheta, Barbara Wollenberg, Friedrich Mrosk, Gabriel Hundeshagen, Maximilian Richter, Max Heiland, Jan Voss, Adriana C. Panayi, Steffen Koerdt, Leonard Knoedler

**Affiliations:** ^1^ School of Medicine, University of Pittsburgh, Pittsburgh, PA, United States; ^2^ Department of Oral and Maxillofacial Surgery, Charité – Universitätsmedizin Berlin, Berlin, Germany; ^3^ Department of Otolaryngology, Head and Neck Surgery, Technical University of Munich (TUM) School of Medicine and Health, Technical University of Munich, Munich, Germany; ^4^ College of Medicine, California Northstate University, Elk Grove, CA, United States; ^5^ Department of Hand, Plastic and Reconstructive Surgery, Burn Center, BG Trauma Center Ludwigshafen, University of Heidelberg, Ludwigshafen, Germany

**Keywords:** extracellular vesicles, head and neck squamous cell carcinoma, immune evasion, therapy resistance, tumor microenvironment

## Abstract

Head and neck squamous cell carcinoma (HNSCC) remains a clinically challenging malignancy due to its intratumoral heterogeneity, aggressive progression, and resistance to multimodal treatment. Extracellular vesicles (EVs)—including exosomes and microvesicles—have gained attention as active contributors to these phenotypes by mediating intercellular signaling and molecular cargo transfer. HNSCC-derived EVs carry oncogenic and drug resistance proteins, along with microRNAs that promote immune evasion and EMT. Enrichment of microRNAs including miR-21, miR-214, and miR-221/222 within EVs supports angiogenesis, apoptosis evasion, and immune suppression. EV-associated PD-L1 impairs antigen presentation and T cell activity, contributing to resistance to checkpoint blockade. Additionally, EVs promote epithelial-to-mesenchymal transition and extracellular matrix remodeling, facilitating invasion and pre-metastatic niche formation. Through modulation of T cell function, macrophage polarization, and stromal recruitment, EVs help establish an immune-tolerant microenvironment. This review synthesizes current knowledge on the mechanistic roles of EVs in HNSCC and discusses their potential as diagnostic biomarkers and therapeutic targets.

## Graphical Abstract

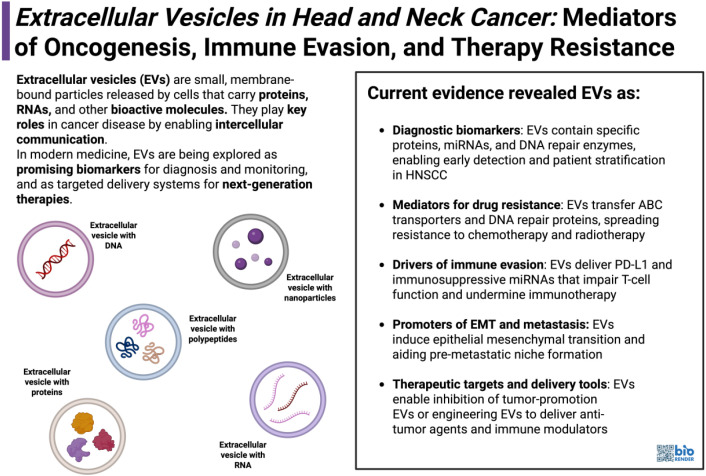

## 1 Introduction

Therapeutic resistance is a pervasive challenge in oncology, accounting for the vast majority of treatment failures in metastatic cancers (1, 2). This resistance emerges from a multifactorial network involving genetic mutations, epigenetic alterations, and microenvironmental influences. Increasingly, extracellular vesicles (EVs) have gained attention as critical mediators of this process (3, 4). These lipid bilayer-enclosed vesicles—secreted by diverse cell types including tumor, stromal, immune, and platelet-derived cells—serve as carriers of bioactive molecules such as proteins, microRNAs, mRNAs, long non-coding RNAs, and fragments of oncogenic DNA (5, 6). By transferring this molecular cargo, EVs facilitate both autocrine and paracrine communication, reprogramming recipient cells and reshaping the tumor microenvironment (TME) to support angiogenesis, immune evasion, metabolic adaptation, and metastasis.

Of particular interest is the role of EVs in undermining anti-tumor immunity and promoting resistance to immunotherapy. In this context, EV-associated PD-L1 can be delivered to T cells and antigen-presenting cells, inducing T cell exhaustion and functional impairment (7–9). Additionally, EVs transport resistance-associated molecules such as ATP-binding cassette (ABC) transporters, anti-apoptotic proteins like Bcl-2, and regulatory microRNAs that collectively suppress apoptosis, enhance DNA repair mechanisms, and facilitate drug efflux (3, 10, 11). These vesicles can also impair antigen presentation and downregulate MHC expression, contributing to the development of an immunologically “cold” tumor microenvironment, even in the presence of immune checkpoint blockade (6, 12, 13).

Head and neck squamous cell carcinoma (HNSCC), a malignancy originating from the mucosal surfaces of the oral cavity, pharynx, and larynx, exemplifies the clinical impact of EV-mediated resistance. Despite aggressive, multimodal treatment strategies—including surgery, radiation, chemotherapy, and immunotherapy—survival rates remain poor for patients with advanced disease (14–16). HNSCC is characterized by a highly immunosuppressive and heterogeneous TME, displaying a spectrum from immune-inflamed to immune-excluded or desert phenotypes (17, 18). These features, coupled with the tumor’s inherent genomic instability and adaptability, make HNSCC a particularly valuable model for studying the functional and translational implications of EVs (17, 19).

**Figure 1:**
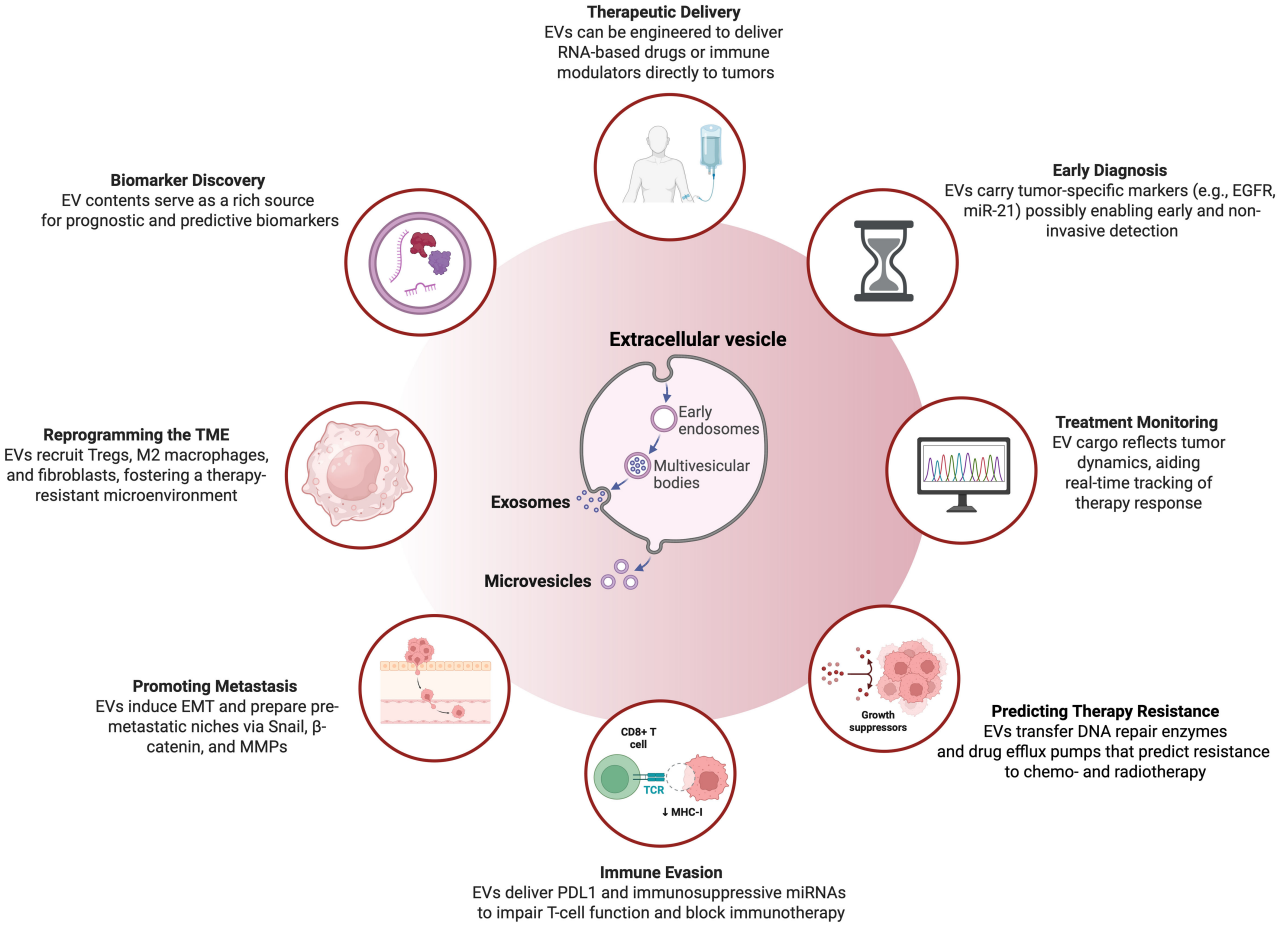
Overview on the multifaceted application and characteristics of extracellular vesicles in head and neck squamous cell carcinoma. This illustration highlights the diverse functions and clinical potential of EVs—primarily exosomes and microvesicles—in HNSCC. Originating from early endosomes and multivesicular bodies, EVs contribute to tumor progression, immune modulation, and therapeutic response. Clinically, they enable early diagnosis via tumor-specific cargo such as EGFR and miR-21, and support treatment monitoring by reflecting dynamic tumor changes. EVs also promote resistance to therapy by transferring DNA repair enzymes and drug efflux pumps, and aid immune evasion through delivery of PD-L1 and immunosuppressive miRNAs. In the tumor microenvironment, they recruit regulatory T cells, M2 macrophages, and fibroblasts, fostering immune suppression and treatment resistance. EVs facilitate metastasis by inducing epithelial-to-mesenchymal transition (EMT) and establishing pre-metastatic niches via Snail, β-catenin, and MMPs. Additionally, they are emerging as delivery platforms for RNA-based drugs and immune modulators, and serve as valuable sources of predictive and prognostic biomarkers. Figure was created using BioRender Premium.

This narrative review aims to synthesize current knowledge on the role of extracellular vesicles in HNSCC, with a focus on their contribution to immune modulation and therapy resistance (**Figure 1**). We begin by outlining EV biogenesis, classification, and cargo profiles (**Table 1**). We then explore the mechanisms by which HNSCC-derived EVs influence tumor progression, immune escape, and resistance to treatment. Finally, we examine emerging therapeutic strategies targeting EVs and discuss future directions for leveraging EV biology to improve outcomes in HNSCC.

**Table 1:** Summary of commonly used techniques for EV isolation and characterization, highlighting key features, strengths, and limitations.

EV Methods	Technique	Key features	Strengths	Limitations	Recommended use
EV Isolation Methods	Differential Ultracentrifugation	Commonly used for EV isolation; relatively simple.	Widely available; cost-effective for large-scale isolation.	Can lead to contamination with non-EV particles; time intensive.	Recommended for bulk EV isolation in research settings where high yield is prioritized over purity.
	Size-Exclusion Chromatography (SEC)	Separates EVs based on size without harsh processing.	Maintains EV integrity; minimal processing artifacts.	Limited scalability; may co-isolate non-EV macromolecules.	Ideal for preserving EV integrity in functional assays and downstream molecular studies.
	Immunoaffinity Capture	Enables selective isolation of specific EV subtypes.	High specificity for targeted EV subpopulations.	Low yield; potential bias in subtype enrichment.	Best suited for targeted studies on specific EV subpopulations or biomarker discovery.
EV Characterization Methods	Nanoparticle Tracking Analysis (NTA)	Measures EV size distribution and concentration using light scattering.	High throughput; provides quantitative data on EV concentration.	Lacks specificity in distinguishing EV subtypes.	Appropriate for routine size and concentration profiling of EV samples.
	Dynamic Light Scattering (DLS)	Provides size distribution based on Brownian motion.	Simple, rapid, and requires minimal sample preparation.	Poor resolution for polydisperse EV populations.	Useful for quick, preliminary size estimation of monodisperse EV samples.
	Transmission Electron Microscopy (TEM)	High-resolution imaging of EV morphology.	Provides detailed visualization of EV structure and morphology.	Requires extensive sample preparation; lacks molecular composition data.	Recommended for morphological validation and structural analysis of isolated EVs.
	Flow Cytometry	Enables single-vesicle analysis of surface markers.	Allows phenotypic characterization of EV subpopulations. This includes specific kits (beads) for exosome surface marker labeling.	The availability and validation of high-quality antibodies against specific EV markers are still limited.	Optimal for identifying and quantifying surface markers on EV subtypes.
	Proteomics, Transcriptomics, Lipidomics	Provides detailed molecular profiling of EV cargo.	High-resolution molecular characterization; identifies EV biomarkers.	Requires high-purity EV preparations and advanced bioinformatics analysis.	Best used in comprehensive omics studies for biomarker discovery or cargo profiling.

These methods vary in specificity, resolution, scalability, and compatibility with downstream analyses, influencing their suitability for different research and clinical applications.

## 2 Extracellular vesicles in HNSCC: mechanisms of tumor progression, immune evasion, and therapeutic resistance

### 2.1 Biogenesis and classification of EVs in HNSCC

EVs in HNSCC include exosomes, microvesicles, and apoptotic bodies, which differ in biogenesis and molecular signature but collectively act as powerful modulators of tumor progression. Exosomes (30–150 nm), originating from multivesicular bodies via ESCRT-dependent and independent pathways, are enriched in HNSCC with markers like ALIX, TSG101, and tetraspanins, and play a central role in immune suppression through PD-L1 delivery (5, 20, 21). Microvesicles (100–1000 nm), formed by membrane budding and cytoskeletal rearrangement, are particularly enriched in chemoresistant HNSCC cells and carry functional efflux pumps such as P-glycoprotein and DNA repair proteins like ERCC1 (3, 4, 17). Apoptotic bodies (1000–5000 nm), once thought to be inert debris, are now recognized as potential vectors for DAMPs and oncogenic DNA, contributing to the evolution of resistance and intratumoral heterogeneity in HNSCC (4, 5, 22–24). In summary, the classification and cellular origin of EVs in HNSCC directly shape their diverse functional roles in therapy resistance, immune modulation, and tumor adaptation (**Table 2**).

**Table 2:** Comparative overview of extracellular vesicle (EV) subtypes.

Feature	Exosomes	Microvesicles (MVs)	Apoptotic bodies
Size Range	30–150 nm	100–1,000 nm	500–2,000 nm
Biogenesis	Intraluminal budding of multivesicular bodies (MVBs); released via exocytosis	Direct outward budding of plasma membrane	Fragmentation of apoptotic cells
Release Trigger	Constitutive or induced by stress	Cellular activation, stress	Programmed cell death (apoptosis)
Markers	CD9, CD63, CD81, TSG101, Alix	ARF6, Annexin A1, Integrins, Selectins	Histones, fragmented DNA, phosphatidylserine
Cargo Composition	Enriched in miRNAs, tRNAs, mRNAs, proteins (e.g., heat shock proteins), lipids	Similar to parent cell; includes proteins, RNAs, lipids	Nuclear fragments, organelles, DNA, and proteins
Function	Intercellular communication, immune modulation, angiogenesis	Signal transmission, coagulation, immune activation	Clearance of cellular debris, immune modulation
Isolation Challenges	Small size complicates separation from lipoproteins	Heterogeneity, overlaps with exosomes	Often mixed with larger EVs or cell debris
Detection Methods	NTA, TEM, Western blot (CD63+, CD81+)	Flow cytometry, DLS, TEM	Microscopy, flow cytometry

This table summarizes key distinguishing features of the three main EV subtypes—exosomes, microvesicles (MVs), and apoptotic bodies. It outlines differences in size range, mechanisms of biogenesis, stimuli for release, surface markers, cargo composition, and biological functions. Additionally, it highlights practical challenges in isolation and commonly used detection methods for each subtype. Understanding these distinctions is critical for accurate characterization and application of EVs in both research and clinical contexts.

### 2.2 Molecular cargo and oncogenic signatures of HNSCC-derived EVs

HNSCC-derived EVs contain a distinctive and functionally potent cargo profile that drives tumor growth and immune modulation. Proteomic analyses confirm enrichment of receptor tyrosine kinases (e.g., EGFR), heat shock proteins (HSP70, HSP90), and ATP-binding cassette transporters (MRP1, ABCG2), all of which support survival signaling and resistance to targeted and cytotoxic therapies (10, 11, 17). In addition, EVs from HNSCC tumors frequently carry DNA repair enzymes such as ERCC1 and XRCC1, enabling recipient cells to better withstand platinum-based chemotherapy (15, 25, 26). Regulatory microRNAs are another key cargo class; miR-21, miR-214, and miR-221/222 are consistently enriched in tumor-derived EVs and modulate gene expression to suppress apoptosis, enhance angiogenesis, and promote invasion (19, 27, 28). These miRNAs exert their effects through well-characterized signaling axes: for example, miR-21 suppresses PTEN, leading to activation of the PI3K-AKT pathway and enhanced cell survival, while miR-221/222 target both PTEN and TIMP3 to promote migration, EMT, and matrix remodeling. In support of this, plasma-derived small EVs (sEVs) from HNSCC patients have been shown to strongly enhance angiogenic potential, underscoring their systemic bioactivity and functional relevance beyond the local tumor microenvironment (29).

### 2.3 EV-mediated mechanisms of resistance in HNSCC

In HNSCC, EVs serve as mobile vectors of resistance, particularly in the context of cisplatin therapy. Exosomes and microvesicles from resistant HNSCC cells are enriched with ERCC1 and XRCC1, which facilitate nucleotide and base excision repair, thereby neutralizing the cytotoxic effects of DNA-damaging agents (15, 17, 30, 31). These vesicles also carry high levels of HSP70 and HSP90, which stabilize DNA repair proteins and stress-response effectors, further enhancing survival under chemotherapeutic pressure (17). Notably, field cancerization in HNSCC allows EVs to transmit resistance phenotypes across spatially distinct tumor foci, fostering a functionally resistant network (4, 5, 26). Moreover, the presence of anti-apoptotic proteins like Bcl-2 in radioresistant HNSCC-derived EVs confirms their role in shielding cells from radiation-induced cell death (32). Overall, EVs in HNSCC orchestrate a multifaceted resistance network that undermines therapeutic efficacy across both clonal populations and anatomical compartments.

### 2.4 EV-induced EMT and metastatic reprogramming in HNSCC

EVs in HNSCC actively drive epithelial-mesenchymal transition (EMT), a key program in metastasis and immune evasion. Tumor-derived EVs transport transcriptional repressors such as Snail and β-catenin, which suppress epithelial markers (e.g., E-cadherin) and upregulate mesenchymal traits (e.g., vimentin, N-cadherin), promoting migratory and invasive capacities (33–36). These EVs also deliver MMP1, MMP3, and integrins (ITGA6, ITGB1), which degrade the extracellular matrix and enable pre-metastatic niche formation—processes particularly relevant in high-grade HNSCC (23, 37). Recent findings have shown that plasma-derived sEVs can reprogram macrophages to facilitate pre-metastatic niche formation in HNSCC, emphasizing their role in priming distant sites for metastatic colonization (38). Furthermore, EVs contain annexins and galectin-3-binding protein (LGALS3BP), which contribute to stromal reprogramming and immune cell dysfunction (39–42). Thereby, EV-driven EMT in HNSCC fosters a dual threat of immune escape and enhanced invasiveness, positioning EVs as central regulators of metastatic evolution.

### 2.5 Immunosuppressive functions of EVs in the HNSCC microenvironment

**Figure 2:**
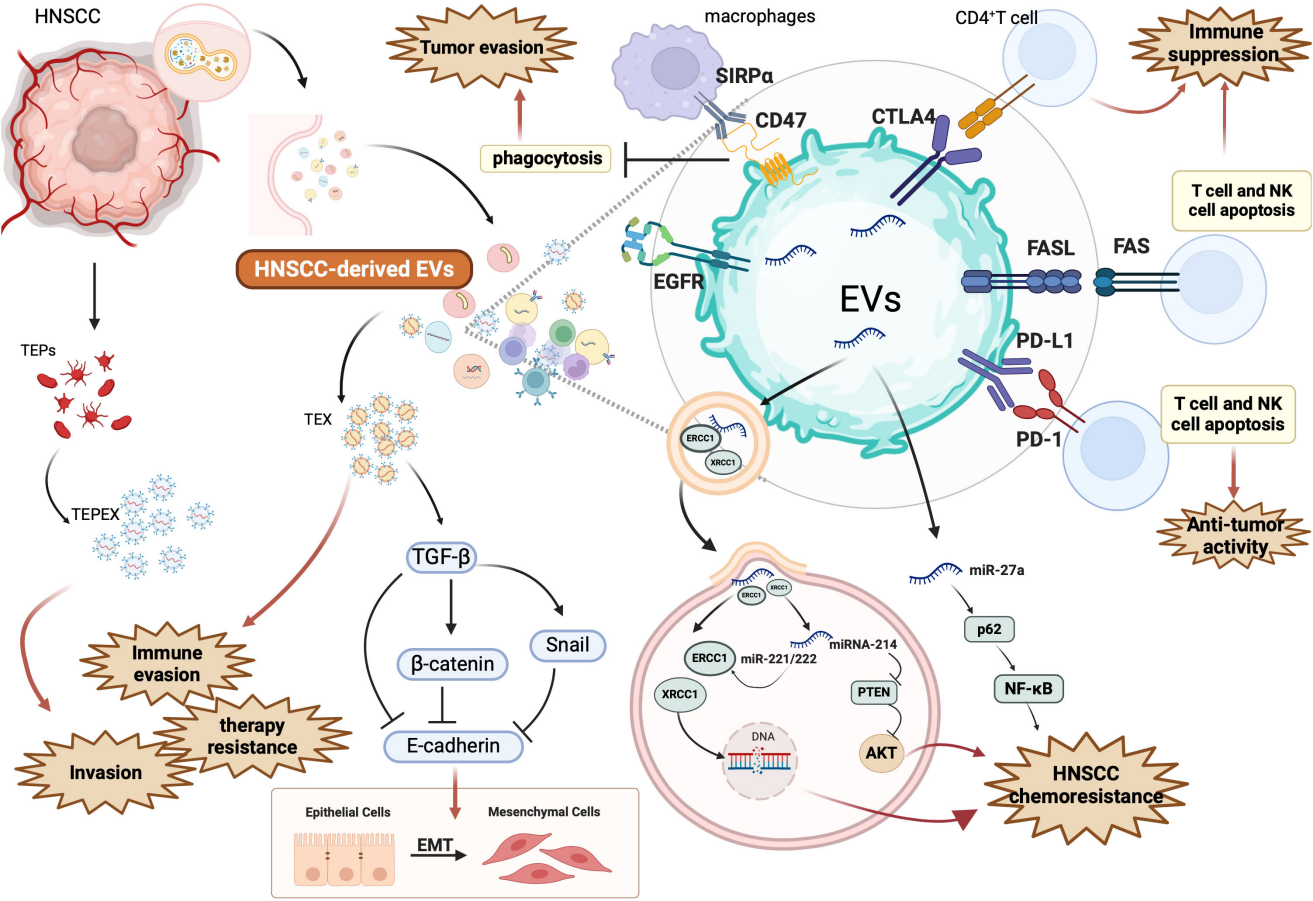
*Mechanisms by which HNSCC-derived EV promote tumor progression, immune suppression, and therapeutic resistance.* This figure illustrates the multifaceted role of EVs secreted by head and neck squamous cell carcinoma (HNSCC) cells in shaping a pro-tumorigenic microenvironment. HNSCC-derived EVs carry a range of immunosuppressive ligands, signaling molecules, and non-coding RNAs that disrupt immune surveillance and enhance cancer cell survival. Specifically, EV-associated PD-L1 and FasL bind to PD-1 and FAS on T cells and NK cells, triggering apoptosis and weakening anti-tumor immunity. They also express CTLA4 and CD47, which inhibit CD4^+^ T cell activation and macrophage-mediated phagocytosis via SIRPα signaling. These EVs also reprogram immune cells and tumor-associated endothelial precursors (TEX, TEPs), contributing to immune evasion and expansion of TEPEX populations. Through cargoes such as TGF-β, β-catenin, and Snail, EVs induce epithelial-to-mesenchymal transition (EMT), characterized by E-cadherin downregulation, thereby promoting invasion and metastasis. EVs further enhance chemoresistance by transferring DNA repair enzymes ERCC1 and XRCC1, and miRNAs such as miR-221/222, miR-214, and miR-27a, which regulate PTEN/AKT and NF-κB pathways. For example, miR-27a suppresses p62, activating NF-κB and supporting resistance. These alterations bolster cell survival and therapy resistance. Additionally, EGFR-enriched EVs may influence receptor-mediated signaling and modulate downstream oncogenic pathways. Collectively, this figure underscores how HNSCC-derived EVs modulate the immune microenvironment, facilitate EMT and invasion, and transmit resistance mechanisms to both immune checkpoint inhibitors and chemotherapies. Figure was created using BioRender Premium.

HNSCC-derived EVs are potent immunosuppressive agents that sculpt a TME conducive to tumor persistence and immune escape. Additionally, miR-27a within these vesicles targets immune-activating genes, suppressing co-stimulatory molecule expression in dendritic cells and macrophages (43, 44). EVs also act as antigen decoys, shedding tumor-associated antigens (TAAs) to divert immune recognition while displaying surface CD47 to inhibit macrophage-mediated clearance (45, 46). Moreover, HNSCC EVs secrete TGF-β and IL-10, driving Treg expansion and M2 macrophage polarization, both of which contribute to a tolerogenic and therapy-resistant microenvironment (6, 13). In summary, EVs play a pivotal role in HNSCC immune evasion by blunting anti-tumor immunity and engineering an immune landscape hostile to therapeutic response. The diverse immunosuppressive, pro-metastatic, and resistance-promoting functions of EVs in HNSCC are summarized in **Figure 2**.

### 2.6 Integrative role of EVs in HNSCC progression and therapeutic resistance

**Figure 3:**
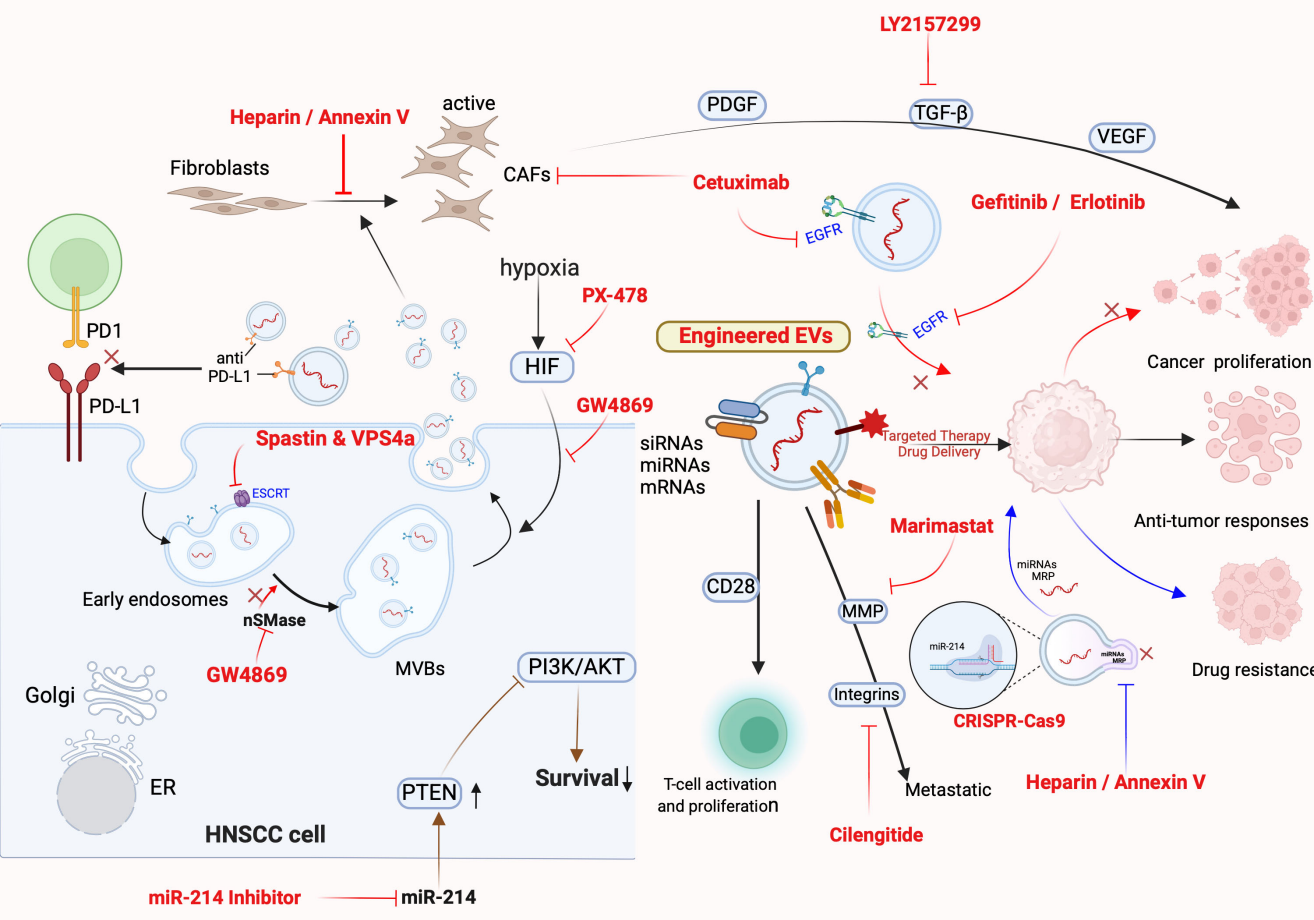
*Therapeutic strategies for targeting EVs in HNSCC.* This figure summarizes new and established strategies aimed at modulating EV biogenesis, release, and function in head and neck squamous cell carcinoma (HNSCC). Pharmacologic agents such as GW4869 (a neutral sphingomyelinase inhibitor) and PX-478 (a hypoxia-inducible factor [HIF] inhibitor) suppress EV production and cargo release under stress conditions, including hypoxia. Spastin and VPS4a, key mediators of multivesicular body (MVB) formation, and the ESCRT machinery, are involved in EV biogenesis and can be therapeutically targeted to reduce vesicle secretion. These approaches remain in the preclinical stage. EV-mediated immune suppression via PD-L1 can be blocked using anti-PD-L1 antibodies (clinically approved), thereby restoring T cell activation through CD28 and reducing tumor immune evasion. Inhibition of miR-214, which regulates PTEN and promotes PI3K/AKT-mediated survival and drug resistance, is another preclinical strategy. Engineered EVs are being developed to deliver therapeutic RNAs (siRNAs, miRNAs, mRNAs) and immune ligands to tumors, aiming to enhance anti-tumor responses and suppress proliferation. Clinically approved agents such as Cetuximab, an anti-EGFR monoclonal antibody, and EGFR tyrosine kinase inhibitors (Gefitinib, Erlotinib) block EGFR-positive EV signaling and downstream cancer growth. TGF-β signaling, which promotes EV-facilitated immunosuppression and cancer-associated fibroblast (CAF) activation, can be targeted with the TGF-β receptor I inhibitor LY2157299 (preclinical). Additional therapies like Marimastat (MMP inhibitor), Heparin/Annexin V (inhibiting EV uptake), and Cilengitide (integrin antagonist) are being explored in preclinical studies to limit metastasis and drug resistance by disrupting EV-ECM and immune interactions. Finally, CRISPR-Cas9 is under investigation as a tool to knock out resistance-related genes or oncogenic miRNAs (e.g., miR-214), offering precision EV-based therapies. Together, these interventions aim to block EV-mediated oncogenic communication, reduce tumor proliferation, enhance immune responses, and overcome chemoresistance. Figure was created using BioRender Premium.

Extracellular vesicles in HNSCC serve as multifaceted conduits of tumor adaptation, orchestrating molecular, cellular, and systemic changes that reinforce malignancy. By transferring oncogenic and resistance-associated cargo, EVs orchestrate interactions between tumor, stromal, and immune compartments that reinforce therapeutic failure (2, 3, 5, 17). Moreover, the capacity of EVs to cross anatomical and histological boundaries through lymphatic and circulatory systems makes them ideal vehicles for intercellular influence across the entire tumor landscape (4, 23, 47). In the vascular compartment, HNSCC-derived EVs have been shown to activate and aggregate platelets through tissue factor in a calcium-dependent manner, potentially facilitating hematogenous metastasis and immune cloaking (48). Strategies to disrupt EV-mediated signaling networks include pharmacologic inhibitors of vesicle secretion, antibodies targeting surface ligands, CRISPR-Cas9-based gene editing, and engineered EVs designed to deliver immunomodulatory or gene-silencing cargo, as illustrated in **Figure 3**.

## 3 Discussion

EVs have emerged as central regulators of tumor biology, playing multifaceted roles in driving oncogenesis, modulating immune responses, and promoting resistance to therapy. In HNSCC, a highly heterogeneous and aggressive malignancy, EVs enable dynamic intercellular communication that reinforces tumor progression and therapeutic failure. This review synthesizes current findings on the contributions of EVs to immune evasion, drug resistance, and metastasis in HNSCC. By elucidating these mechanisms, new avenues may emerge for leveraging EVs as diagnostic biomarkers and therapeutic targets in this challenging disease.

Our review found EVs in HNSCC to play a pivotal role in tumor progression by transferring oncogenic proteins, drug resistance factors, and immunosuppressive molecules, thereby promoting chemoresistance, immune evasion, and metastasis. Their ability to drive epithelial-mesenchymal transition, remodel the tumor microenvironment, and disseminate resistance traits across tumor sites highlighted their integrative function in maintaining malignant phenotypes.

However, despite the growing understanding of EV-mediated oncogenesis, literature revealed several challenges in studying and therapeutically targeting EVs. The heterogeneity of EV populations, the dynamic nature of their biogenesis, and the diversity in their molecular cargo present substantial obstacles in precisely defining their functional roles (12, 47). Additionally, the lack of standardized methods for EV isolation, characterization, and functional analysis continues to hinder the translation of laboratory findings into clinical applications (49, 50). Several recent advances are helping to overcome the technical challenges posed by EV heterogeneity and lack of standardized workflows. Updated protocols from the International Society for Extracellular Vesicles (MISEV) now provide widely adopted guidelines for EV nomenclature, isolation, and characterization, helping to reduce methodological variability across studies (51). On the technical front, bead-based multiplex assays and immunocapture platforms now allow for parallel profiling of distinct EV subpopulations from patient plasma or cell culture media (52). In addition, tools like tangential flow filtration (TFF), size-exclusion chromatography, and the exoRNeasy system have improved reproducibility and purity across isolation protocols (53). Single-EV profiling using nano-flow cytometry or super-resolution microscopy is also increasingly accessible, allowing researchers to resolve cargo heterogeneity within individual vesicles (54).

The immunomodulatory effects of EVs, particularly through PD-L1 delivery, further complicate the evolving landscape of immunotherapy in HNSCC. EV-mediated PD-L1 transfer enhances immune evasion by suppressing T-cell activity, creating an immunosuppressive microenvironment that favors tumor persistence and therapeutic resistance (7–9). The challenge of intercepting EV-driven immune escape necessitates novel strategies that restore immune surveillance and enhance the efficacy of immune checkpoint inhibitors.

Despite these challenges, targeting EV biogenesis, release, and uptake presents critical focus of ongoing translational research. Engineered EVs that selectively deliver anti-tumor agents, inhibitors of oncogenic pathways, or immune-modulating molecules offer a precision medicine approach to counteract EV-mediated disease progression, although therapeutic specificity remains complicated by EV heterogeneity (3, 4). Additionally, small-molecule inhibitors that block EV formation or disrupt their interactions with recipient cells could enhance the efficacy of existing treatments by mitigating drug resistance and limiting metastatic spread (5, 49). Heterogeneity presents a translational challenge because differences in vesicle origin, cargo, and surface markers complicate efforts to selectively target tumor-promoting subsets while preserving normal intercellular signaling. New technologies such as single-vesicle flow cytometry, high-resolution proteomics, and droplet-based microfluidics are enhancing the sensitivity and resolution of EV profiling, offering new avenues for dissecting EV subtypes and improving biomarker discovery (55–57). As research advances, the development of EV-targeted therapeutics and EV-based biomarkers has the potential to revolutionize cancer diagnostics and treatment paradigms (3, 5, 13).

This review underscores EVs as mediators of immune evasion, therapeutic resistance, and metastatic behavior in HNSCC. For clinicians, the translational potential lies in leveraging EVs as diagnostic biomarkers, such as for PD-L1 or drug-resistance markers and as novel therapeutic targets, including strategies that block EV secretion, uptake, or immunosuppressive cargo. Arginase-1 enrichment in plasma-derived EVs has been identified as a potential biomarker for metastatic disease in HNSCC patients, alongside other clinically investigated EV markers such as PD-L1, EGFR, and miR-21, offering minimally invasive tools for risk stratification and therapy response monitoring (58).

In addition to Arginase-1, several other EV-based applications are entering clinical workflows. PD-L1–expressing EVs have been proposed as predictive biomarkers to stratify patients for immune checkpoint inhibitor therapy, particularly in non-responders with low tumor cell PD-L1 expression but high EV-PD-L1 burden (59). Moreover, liquid biopsies leveraging EV cargo such as miR-21 and EGFR have shown promise in early detection and recurrence monitoring, with prospective trials exploring their integration into routine follow-up protocols (60). Therapeutically, engineered EVs are being developed to deliver targeted siRNAs, CRISPR-Cas9 systems, or immune ligands to tumor sites, enabling cell-type–specific modulation with minimal systemic toxicity (34). For example, miR-34a has shown potent anti-tumor effects in HNSCC xenograft models when delivered using chemically stabilized mimics, and its therapeutic delivery via engineered EVs is currently under investigation in other cancer types, supporting its potential for EV-based applications in HNSCC (61).

EVs from HNSCC are increasingly studied for liquid biopsy. For example, circulating exosomal PD‐L1 levels correlate with tumor stage and can predict relapse (62). Similarly, tumor‐derived exosomal EGFR and phospho‐EGFR fall during anti‐EGFR (cetuximab) therapy, suggesting EV‐EGFR can monitor therapeutic response (63). EV‐microRNAs also show promise: an 11‐miRNA signature in serum EVs robustly detected HPV^+^ oropharyngeal SCC (64). On the therapy side, EVs can be engineered as drug carriers. Engineered EVs have been loaded with siRNAs, cytokines or even gene‐editing systems (e.g., CRISPR/Cas9) to modulate tumors (65, 66). For instance, exosomes displaying immunostimulatory ligands or checkpoint inhibitors have been constructed to activate T cells (7, 67). Several clinical trials now involve EV platforms. For instance, a Phase I trial (NCT03608631) is testing MSC‐derived exosomes delivering KRAS‐G12D siRNA in pancreatic cancer (68). In fact, multiple EV‐based trials (most using MSC or dendritic‐cell EVs) are registered for solid tumors (69).

However, clinical translation and translation to HNSCC in particular faces key hurdles: the heterogeneity of EV populations, lack of standardization in isolation and profiling methods, and incomplete understanding of their biological roles all limit current applicability. Furthermore, selectively targeting tumor-promoting EVs without disrupting physiological intercellular communication remains a major challenge. For patients, the promise of EV-based interventions is substantial, offering hope for more personalized and effective therapies—especially in refractory or recurrent disease—but realization of these benefits will depend on robust clinical validation. Overall, future research must focus on refining EV detection technologies, validating EV-based biomarkers in large patient cohorts, and rigorously testing EV-targeted therapies in preclinical and clinical models.

## 4 Conclusion

By shaping the tumor microenvironment, promoting immune escape, and facilitating metastasis, EVs serve as key drivers of therapeutic resistance and disease persistence. Their biofluid stability, tumor-derived cargo, and immunomodulatory roles make EVs promising candidates for precision diagnostics and therapeutic targeting in HNSCC. However, clinical translation is limited by the heterogeneity of EV populations, lack of standardization in isolation and characterization methods, and incomplete understanding of their context-specific functions. Future work should focus on refining EV profiling techniques, developing selective targeting strategies, and validating clinical applications in well-designed studies. As our understanding deepens, EVs may ultimately offer a novel framework for precision diagnostics and therapy in HNSCC.

## Glossary

ABC: ATP-binding cassette
ABCG2: ATP-binding cassette sub-family G member 2
ALIX: Apoptosis-linked gene 2-interacting protein X
BER: Base excision repair
Bcl-2: B-cell lymphoma 2
CAF: Cancer-associated fibroblast
DAMP: Damage-associated molecular pattern
DLS: Dynamic light scattering
EGFR: Epidermal growth factor receptor
ECM: Extracellular matrix
ESCRT: Endosomal sorting complex required for transport
EV: Extracellular vesicle
HIF: Hypoxia-inducible factor
HNSCC: Head and neck squamous cell carcinoma
HSP: Heat shock protein
IL-8: Interleukin-8
ITGA6: Integrin alpha-6
ITGB1: Integrin beta-1
LGALS3BP: Galectin-3-binding protein
lncRNA: Long non-coding RNA
LAMP1: Lysosomal-associated membrane protein 1
LAMP2: Lysosomal-associated membrane protein 2
MAPK: Mitogen-activated protein kinase
MMP: Matrix metalloproteinase
MRP1: Multidrug resistance protein 1
MVB: Multivesicular body
mRNA: Messenger RNA
NER: Nucleotide excision repair
NTA: Nanoparticle tracking analysis
PD-1: Programmed cell death protein 1
PD-L1: Programmed death-ligand 1
PDCD6IP: Programmed cell death 6-interacting protein
PI3K/AKT: Phosphoinositide 3-kinase/Protein kinase B
PS: Phosphatidylserine
PTEN: Phosphatase and tensin homolog
SEC: Size-exclusion chromatography
SSB: Single-strand break
siRNA: Small interfering RNA
TAA: Tumor-associated antigens
TGF-β: Transforming growth factor beta
TIL: Tumor-infiltrating lymphocyte
TME: Tumor microenvironment
TNM: Tumor-node-metastasis classification system
TSG101: Tumor susceptibility gene 101
VEGF: Vascular endothelial growth factor
VIM: Vimentin
XRCC1: X-ray repair cross-complementing protein 1.
